# Comparing performance of mothers using simplified mid-upper arm circumference (MUAC) classification devices with an improved MUAC insertion tape in Isiolo County, Kenya

**DOI:** 10.1186/s13690-018-0260-x

**Published:** 2018-02-22

**Authors:** Angeline Grant, James Njiru, Edgar Okoth, Imelda Awino, André Briend, Samuel Murage, Saida Abdirahman, Mark Myatt

**Affiliations:** 1 Action Against Hunger, One Whitehall St, New York, NY 10004 USA; 2 Action Against Hunger, Nyangumi Road, PO Box, Nairobi, 39900–00623 Kenya; 3 Department of International Health, University of Tampere School of Medicine, PB 100 Tampere, Finland; 4 Department of Nutrition, Exercise and Sports, Faculty of Science, University of Copenhagen, DK-1958 Frederiksberg, Denmark; 5 National Ministry of Health Unit of Nutrition, Monitoring and Evaluation Department, Old Mbagathi Road, PO Box 43319–01000, Nairobi, Kenya; 6 Isiolo County Health Management Team, Hospital Road, PO Box 36–30600, Isiolo, Kenya; 7 Brixton Health, Alltgoch Uchaf, Llawryglyn, Caersws, Powys SY17 5RJ UK

**Keywords:** Screening by mothers, Severe acute malnutrition, Community management of acute malnutrition, Mid-upper arm circumference

## Abstract

**Background:**

A novel approach for improving community case-detection of acute malnutrition involves mothers/caregivers screening their children for acute malnutrition using a mid-upper arm circumference (MUAC) insertion tape. The objective of this study was to test three simple MUAC classification devices to determine whether they improved the sensitivity of mothers/caregivers at detecting acute malnutrition.

**Methods:**

Prospective, non-randomised, partially-blinded, clinical diagnostic trial describing and comparing the performance of three “Click-MUAC” devices and a MUAC insertion tape. The study took place in twenty-one health facilities providing integrated management of acute malnutrition (IMAM) services in Isiolo County, Kenya. Mothers/caregivers classified their child (*n*=1040), aged 6–59 months, using the “Click-MUAC” devices and a MUAC insertion tape. These classifications were compared to a “gold standard” classification (the mean of three measurements taken by a research assistant using the MUAC insertion tape).

**Results:**

The sensitivity of mother/caregiver classifications was high for all devices (>93% for severe acute malnutrition (SAM), defined by MUAC < 115 mm, and > 90% for global acute malnutrition (GAM), defined by MUAC < 125 mm). Mother/caregiver sensitivity for SAM and GAM classification was higher using the MUAC insertion tape (100% sensitivity for SAM and 99% sensitivity for GAM) than using “Click-MUAC” devices. Younden’s *J* for SAM classification, and sensitivity for GAM classification, were significantly higher for the MUAC insertion tape (99% and 99% respectively). Specificity was high for all devices (>96%) with no significant difference between the “Click-MUAC” devices and the MUAC insertion tape.

**Conclusions:**

The results of this study indicate that, although the “Click-MUAC” devices performed well, the MUAC insertion tape performed best. The results for sensitivity are higher than found in previous studies. The high sensitivity for both SAM and GAM classification by mothers/caregivers with the MUAC insertion tape could be due to the use of an improved MUAC tape design which has a number of new design features. The one-on-one demonstration provided to mothers/caregivers on the use of the devices may also have helped improve sensitivity. The results of this study provide evidence that mothers/caregivers can perform sensitive and specific classifications of their child’s nutritional status using MUAC.

**Trial registrations:**

Clinical trials registration number: NCT02833740

## Background

It is currently estimated that, at any one time, over 17 million children under the age of five years suffer from severe acute malnutrition (SAM) [1], possibly translating to more than 100 million global incident cases each year [2]. Over the past two decades there has been a shift from an in-patient, hospital-based treatment approach for SAM to a decentralised model combining both out-patient care for uncomplicated cases of SAM and in-patient care for SAM children with medical complications or those not responding to treatment [3]. Uncomplicated cases of SAM are treated in an out-patient therapeutic programme (OTP) while complicated cases of SAM are medically stabilised in a nutrition stabilisation centre before being referred for out-patient care in the OTP. This model, known as community management of acute malnutrition (CMAM), or integrated management of acute malnutrition (IMAM) in some contexts, has significantly increased the number of SAM cases receiving treatment in recent years. However, despite these gains, it has been estimated that less than 20% of SAM children are currently accessing treatment globally [4].

A key component of CMAM is ensuring regular screening and case-finding at community level. Since the scaling up of CMAM, mid-upper arm circumference (MUAC) measurement has become the most common form of anthropometric measurement used at community and primary health centre level for the case-finding and admission of cases of acute malnutrition. Most acute malnutrition case-finding is carried out by community health workers (CHW) or community health volunteers (CHV) who measure MUAC and refer children with a MUAC of less than 115 mm for therapeutic feeding and medical care [5–7]. Children with a MUAC of less than 125 mm are referred for supplementary feeding support if this is available. MUAC has been shown to be the best prognostic indicator for mortality in children aged 6–59 months [8–13], especially when repeated over time [14] and has been demonstrated to be a safe and effective anthropometric criterion for diagnosis of acute malnutrition and admission for acute malnutrition treatment [15–19]. If a SAM case is detected and acted upon early in the disease episode this can decrease mortality and morbidity related to malnutrition, reduce per-case treatment costs thanks to shorter treatment times and lower the numbers of children requiring expensive in-patient care for SAM with medical complications [20, 21]. A combination of high cure rates and short treatment lengths often acts to increase SAM treatment programme coverage [8].

A novel community screening approach involves mothers and caregivers using MUAC to detect acute malnutrition in their own children [22]. This may enable mothers and caregivers to develop a better understanding of the signs of malnutrition, be engaged in monitoring their children’s nutrition status and increase the frequency of child screening at community level.

A study conducted in Niger in 2013–2014 [23] demonstrated a significantly higher median MUAC at admission to OTP and better OTP Sphere standards performance indicators [24] in areas where mothers were screening their own children compared to areas where CHWs were responsible for screening children. The study also showed lower proportions of children needing in-patient care at admission or during treatment and reduced numbers of rejected referrals (i.e. children who did not fulfil OTP entry criteria of MUAC < 115mm - an important barrier to coverage [25]) in areas where mothers did the MUAC screening. The coverage of the OTP in the areas where the mothers did the screening was comparable to coverage of the OTP in the areas where the CHWs did the screening.

The work carried out to date on supporting mothers to measure MUAC is based on the utilisation of conventional MUAC tapes. These tapes are colour-coded and/or graduated. They are made of flexible material (e.g. polypropylene or plasticised paper) about 1 cm wide. As per international guidelines [26] the MUAC tape is placed on the middle of the left upper arm of the child. The tension of the band is adjusted by the person undertaking the measurement. Errors with too tight or too loose tape measurements can be observed. Measurement error may decrease the sensitivity of the diagnosis. A previous study conducted in rural Niger [27] found that mothers could use colour-banded MUAC tapes to identify cases of SAM (defined by MUAC < 115 mm) with 73% sensitivity and 98% specificity. It was therefore proposed to develop three simplified and standardised (either 115 mm circumference or including both 115 mm and 125 mm circumferences) MUAC bracelets (“Click-MUAC” devices) to support the mother-led MUAC screening approach and to test these prototypes in an operational setting. The prototypes would be compared to a universal design (i.e. for use with adults, children, and neonates for chest and head circumference), colour-banded MUAC insertion tape (“uniMUAC”). The uniMUAC tape is a modified design (i.e. to improve accuracy) compared to existing models such as the UNICEF MUAC tape [28]. The uniMUAC tape has shown increased accuracy and similar precision, when compared to conventional design MUAC tapes, in tests using soft plastic tubes of known circumference (between 100 mm and 160 mm) [29].

The primary aim of the study was to describe and compare the performance of a set of prototype “Click-MUAC” devices against a “gold standard” of classification, in terms of five measures (sensitivity, specificity, agreement, Fleiss’ *Kappa* and Youden’s *J*), for the classification (diagnosis) of nutritional status (SAM, moderate acute malnutrition, normal).

The secondary aim of the study was to determine the difference in agreement with the “gold standard” classification amongst mothers/caregivers using “Click-MUAC” devices versus mothers/caregivers using a MUAC insertion tape.

## Methods

**Fig. 1:**
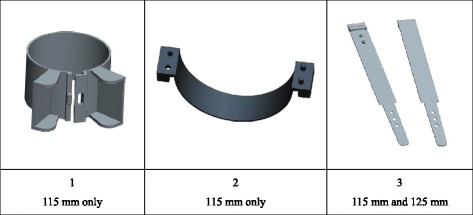
The three “Click-MUAC” prototypes used in the study. Devices 1 and 2 have an internal circumference of 115 mm. Device 3 has an internal circumference of either 115 mm or 125 mm depending on how the device is latched

Three “Click-MUAC” devices and one MUAC insertion tape were used in the study. The “Click-MUAC” devices were developed with the support of nutrition specialists, plastics specialists and 3D-printing experts in France, the United Kingdom, the United States and Kenya. Brainstorming around product design was initially supported with 3D-printed prototypes, one of which was subsequently pursued by a separate research team [30]. However 3-D printing was not retained as the final prototype manufacturing process for the study, as the designs chosen required a level of detail, functionality and robustness that necessitated a more complex production process. Eighteen prototypes (six specimens of each of the three “Click-MUAC” designs) were produced by a plastics manufacturing company using a plastic printing injection process [31]. This process involved the injection of polypropylene into a resin mould to create functional semi-rigid prototypes. The tolerance and repeatability of the final prototypes were assessed and deemed by the manufacturer and the study team to be similar across the eighteen specimens. The “Click-MUAC” prototypes had a standard measurement of 115 mm (prototypes 1 and 2 – see Fig. 1) and 115 mm and 125 mm (prototype 3 – see Fig. 1).

**Fig. 2:**
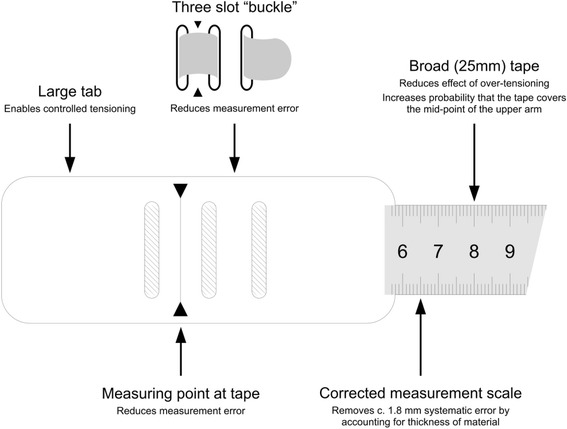
Features of the universal design MUAC insertion tape used in the study

The study also used a universal design, colour-banded MUAC insertion tape (“uniMUAC”), with 1 mm graduation, designed and produced by a consortium of Non-Governmental Organisations and academics led by Médecins Sans Frontières. The universal MUAC tape was designed to minimise measurement error by having a large tab to enable controlled tensioning of the tape; a three slot "buckle" to hold the tape straight while measurements are taken; a broad tape to reduce the effect of over-tensioning and to increase the probability that the tape covers the mid-point of the upper arm; measuring points that extend to the edge of the tape; and a corrected measurement scale to remove a systematic error of at least +1.8 mm in MUAC measurements found in other conventional design MUAC tapes, which is due to a failure to account for the thickness of the tape material when positioning the scale and/or measurement point. These design elements are shown in Fig. 2.

The study was conducted according to the Declaration of Helsinki guidelines and is registered at clinicaltrials.gov (Trial number: NCT02833740). The study protocol was granted ethical approval by the African Medical and Research Foundation (AMREF) Ethics and Scientific Review Committee, Kenya (ESRC number P249/2016) in July 2016.

Superiority was defined as an increase in case-finding sensitivity of 15% or more accompanied by little or no change in specificity. It was calculated that a sample size of *n* = 115 for each of the three groups (i.e. SAM, moderate acute malnutrition and normal) would be sufficient to determine superiority with better than 95% power, with one-sided *p* = 0.05, and was feasible to collect in the study programme.

The study took place in Isiolo County, Kenya. Action Against Hunger has been active in Isiolo County since 2009, supporting nutrition-specific and nutrition-sensitive programming in collaboration with the Isiolo County Management Team. The county offered IMAM services, supported by Action Against Hunger, where the devices could be tested.

The study was carried out by the Isiolo County Health Management Team (CHMT), with support from Action Against Hunger. Eight research assistants were trained by the CHMT and Action Against Hunger on the study protocol, interview techniques, obtaining informed consent and detection of acute malnutrition through the measurement of MUAC and testing for oedema. Two measurement standardization tests were conducted and analysed using Emergency Nutrition Assessment software (July 9, 2015 version) in order to verify the accuracy and precision of the measurements taken by the research assistants, following the Habicht method [32]. The questionnaire was pre-tested by the study team on patients at Isiolo County Hospital, prior to starting data collection. Sensitisation on the study, its protocol and its objectives, was also provided to participating health facility staff by the CHMT prior to starting data collection. This helped to facilitate the integration of the study into the routine screening activities conducted within the Isiolo County IMAM programme.

Data collection took place in 7 health facilities offering IMAM services. The selected sites were high-caseload facilities and were chosen in order to be able to test the prototypes on a large number of children with SAM or moderate acute malnutrition (MAM). The selection of the health facilities was done by the CHMT, based on county health records. Data collection took place from the 26th of September 2016 to the 26th of January 2017.

During data collection the health facility staff described the study and presented the prototypes to mothers and caregivers during health sensitisation sessions, which took place early in the morning, before the start of clinic appointments. Thereafter, the data collection team discussed the study individually with mothers and caregivers of children aged 6–59 months (the standard age range for IMAM services) who were entering triage at the health facility. The data collection team provided mothers and caregivers with information on the study, what participation entailed, the risks and benefits of participation and how data confidentiality would be maintained. The mothers/caregivers who agreed to participate, and whose children met the inclusion criteria, were asked to provide consent. Consent was obtained by the data collection team in either written or verbal form and was recorded through signature or thumb prints on individual consent forms. The consent forms that recorded consent through thumb prints were signed by a literate witness with no connection to the study team. The witness was often a community health volunteer (CHV) known to the mother/caregiver. Those not known to the CHV had their thumb prints witnessed by a literate mother who happened to be at the same health facility that day. Children enrolled in the study were mainly children starting or already receiving IMAM treatment services, children visiting the paediatric outpatient department, children attending the child welfare clinic or children whose mothers were involved in mother-to-mother support group meetings for infant and young child feeding at the health facility.

Once a child was enrolled, the data collection team collected identifying and demographic data and then demonstrated the use of the 3 “Click-MUAC” devices and the colour-banded MUAC insertion tape to the mother/caregiver. The mother/caregiver then classified her child’s nutrition status with the 3 “Click-MUAC” devices and the colour-banded MUAC insertion tape. These 4 classifications were recorded by the data collection team. The recorded data was then obscured (by means of a folding data collection form) allowing for partial blinding of the results. The series of MUAC classifications was then repeated by the health facility staff. These 4 health facility staff classifications were also recorded by the data collection team and obscured by folding the data collection form a second time. Mothers/caregivers and health facility staff were also asked to identify their preferred device.

The data collection team then took 3 measurements of the child’s MUAC with the colour-banded MUAC insertion tape at the measured mid-point of the left arm of each study subject. Classifications were made by comparing the arithmetic mean of the three measurements (i.e. the “gold standard” measurement) against case-defining thresholds for global acute malnutrition (i.e. MUAC < 125 mm) and severe acute malnutrition (i.e. MUAC < 115 mm). Any child identified as SAM or MAM, who was not already enrolled in the IMAM programme, was referred for IMAM services.

A mid-term review of the data collection process in December 2016 highlighted that the SAM case numbers were lower than had been expected. To ensure that the SAM sample size (*n* = 115) was reached, data collection was expanded to an additional 14 facilities and community-based case-finding was strengthened.

Collected data were entered into a purpose-designed EpiData v3.1 database [33]. Data were checked for range and legal values during data-entry. Data were double-entered and validated with discrepancies resolved by reference to data collection forms.

Five measures (sensitivity, specificity, agreement, Fleiss’ *Kappa*, and Youden’s *J*) for the different measurer groups (i.e. study staff, clinic staff, and mothers/caregivers) with regard to MUAC classification were calculated from two-by-two contingency tables. Sensitivity was defined as the ability of a device to correctly detect patients with the condition (SAM or MAM), specificity was defined as the ability of a device to correctly detect patients without the condition (SAM or MAM) and agreement was defined as the proportion of cases where the classification was the same as that of the “gold standard”. Fleiss’ *Kappa* [34] and Younden’s *J* [35] are both chance-corrected measures of agreement.

Data were analysed using purpose-written *R* language scripts managed using the *R-AnalyticFlow* scientific workflow system [36, 37]. Bootstrap methods were used to calculate 95% confidence intervals on summary statistics using *r* = 9999 replicates. Exact binomial confidence limits were calculated in two cases where 100% sensitivity was observed.

## Results

Table 1 shows the description of the study sample. The total sample size for the study was 1040 children. The minimum sample (i.e. *n* = 115) was reached for each of three groups (i.e. MUAC ≥ 125 mm; 115 mm ≤ MUAC < 125 mm; and MUAC < 115 mm). The majority of children enrolled in the study came from the paediatric outpatient appointments (61.4%), followed by children attending the child welfare clinic or those whose caregivers were participating in infant and young child feeding support (29.3%). The distribution of the “gold standard” measure (i.e. the mean of 3 measurements of the MUAC as taken by a research assistant) ranged from 86 mm to 190 mm, with a median of 137 mm. Within-subject differences between the three MUAC measurements used to create the "gold standard" classification were investigating by comparing all possible pairs of within subject-measurements (*n* = 3120 measurements). It was found that 3075 (98.6%) differences were less than or equal to 2 mm. The maximum difference found was 4 mm, present in 3 (0.01%) of measurements. The mean difference was close to zero (mean = 0.0032, SD = 1.0227).

**Table 1:** Description of the study sample

Item	Group	Number	Percentage
Sample size	All children	1040	100.0%
Sex of child	Females	513	49.3%
	Males	527	50.7%
MUAC class^a^	MUAC ≥ 125 mm	698	67.1%
	115 mm ≤ MUAC < 125 mm	217	20.9%
	MUAC < 115 mm	125	12.0%
Source	OTP or SFP program	96	9.2%
	Paediatric outpatients	639	61.4%
	Other source	305	29.3%
Item	Summary	Value	Units
Age of child	Minimum	6	Months
	Lower quartile	11	
	Median	18	
	Upper quartile	29	
	Maximum	59	
	Mean (SD)	21.37 (13.0)	
MUAC^b^	Minimum	86	Mm
	Lower quartile	123	
	Median	137	
	Upper quartile	148	
	Maximum	190	
	Mean (SD)	136 (16.6)	

^a^Case-definitions applied to the mean of 3 measurements taken by a research assistant

^b^Mean of 3 measurements taken by a research assistant

**Fig. 3:**
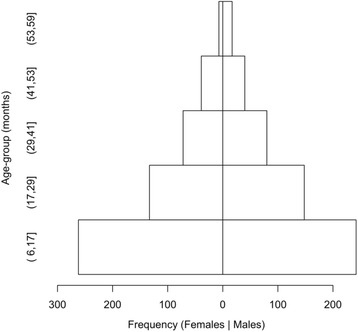
Age and sex distribution of the study sample. Ranges are expressed in ISO 31–11 form [A] The form (a,b] expresses the interval a < x ≤ b. For example, (17,29] is used to indicate the set {18, 19, 20, 21, 22, 23, 24, 25, 26, 27, 28, 29} of ages in months. Age-heaping, the tendency of respondents to report ages round to the nearest year or six months is common in many settings. This is a major reason why data from nutritional surveys and programs are often analysed and reported using broad age-groups. The commonest age-groups used with children’s data are 6 to 17 months, 18 to 29 months, 30 to 41 months, 42 to 53 months, and 54 to 59 months. These are known as year-centred age-groups. The last age-group covers only six months but is nominally centred at five years.

Figure 3 shows the age and sex distribution of the study sample. The distribution of ages was similar for males and females (Chi-square = 6.0074, df = 4, p = 0.1986).

Table 2 shows that for SAM classification by mothers, compared to the “gold standard” measurement, all three “Click-MUAC” devices demonstrated good sensitivity (>93%) and excellent specificity (>98%). The chance-corrected measure of agreement (Younden’s *J*) between mothers’ classification for SAM with the “Click-MUAC” devices and the “gold standard” was also high (>92%). Prototype 3 performed the best out of the 3 “Click-MUAC” devices with a sensitivity of 96.1% [95% CI 92.3%; 99.2%] compared to the “gold standard”. However the device that produced the most sensitive classification (100.0% [95% CI 97.1%; 100.0%]) for mothers, with the highest level of agreement (98.9% [95% CI 98.3%; 99.5%]), was the MUAC insertion tape (device 4). The difference in agreement between prototype 3 and the MUAC insertion tape is statistically significant for Younden’s *J*: 94.9% [95% CI 91.0%; 97.9%] versus 98.8% [95% CI 98.0%; 99.5%].

**Table 2:** Results for “Click-MUAC” devices and uniMUAC tape used by mothers/caregivers and clinic staff compared to case-definition applied to the mean of 3 MUAC measurements taken by a research assistant

	Test	Device^b^	Sensitivity^c^	Specificity^c^	Agreement^c^	*Kappa* ^c^	Youden’s *J*^c^
SAM^d^	MSD^a^	1	93.7% (89.0%, 97.5%)	98.8% (98.0%, 99.5%)	98.2% (97.3%, 98.9%)	0.92 (0.87, 0.95)	92.5% (87.6%, 96.4%)
		2	93.8% (89.1%, 97.5%)	98.7% (97.9%, 99.4%)	98.1% (97.2%, 98.9%)	0.91 (0.87, 0.95)	92.4% (87.7%, 96.3%)
		3	96.1% (92.3%, 99.2%)	98.8% (98.0%, 99.5%)	98.5% (97.7%, 99.1%)	0.93 (0.89, 0.96)	94.9% (91.0%, 97.9%)
		4	100.0% (97.1%, 100.0%)	98.8% (98.0%, 99.5%)	98.9% (98.3%, 99.5%)	0.95 (0.92, 0.98)	98.8% (98.0%, 99.5%)
	CSD^a^	1	92.1% (86.9%, 96.4%)	99.1% (98.5%, 99.7%)	98.3% (97.4%, 99.0%)	0.92 (0.88, 0.95)	91.3% (86.0%, 95.6%)
		2	94.6% (90.1%, 98.2%)	98.9% (98.2%, 99.6%)	98.4% (97.5%, 99.1%)	0.92 (0.88, 0.96)	93.5% (89.0%, 97.0%)
		3	96.1% (92.1%, 99.2%)	99.0% (98.3%, 99.6%)	98.7% (97.9%, 99.3%)	0.94 (0.90, 0.97)	95.1% (91.1%, 98.1%)
		4	100.0% (97.1%, 100.0%)	99.2% (98.7%, 99.8%)	99.3% (98.9%, 99.8%)	0.97 (0.94, 0.99)	99.2% (98.7%, 99.8%)
GAM^d^	MSD^a^	3	90.7% (87.4%, 93.7%)	96.2% (94.6%, 97.5%)	94.3% (92.9%, 95.7%)	0.87 (0.84, 0.90)	86.8% (83.2%, 90.1%)
		4	99.1% (98.0%, 100.0%)	96.5% (95.0%, 97.7%)	97.3% (96.3%, 98.3%)	0.94 (0.92, 0.96)	95.6% (93.7%, 97.2%)
	CSD	3	91.8% (88.9%, 94.6%)	97.2% (95.8%, 98.3%)	95.4% (94.0%, 96.6%)	0.89 (0.86, 0.92)	89.0% (85.6%, 92.0%)
		4	98.0% (96.4%, 99.4%)	97.4% (96.2%, 98.5%)	97.6% (96.6%, 98.5%)	0.95 (0.92, 0.97)	95.4% (93.4%, 97.2%)

^a^MSD = Classification by mother/caregiver made using the specified “Click-MUAC” device or uniMUAC tape; CSD = Classification by IMAM clinical staff using the specified “Click-MUAC” device or uniMUAC tape

^b^Numbers 1, 2 and 3 refer to specific “Click-MUAC” devices. Device 4 is the uniMUAC tape

^c^Point estimates and associated 95% confidence intervals of summary measures are reported

^d^SAM is defined as MUAC < 115 mm; GAM is defined as MUAC < 125 mm. Devices 1 and 2 did not allow for GAM assessment

SAM classification by clinic staff using the three “Click-MUAC” devices, compared to the “gold standard”, was good (sensitivity > 92%, specificity > 98%, Younden’s *J* > 91%) however the MUAC insertion tape performed better with a sensitivity of 100.0% [95% CI 97.1%; 100.0%], a specificity of 99.2% [95% CI 98.7%; 99.8%] and a Younden’s *J* of 99.2% [95% CI 98.7%; 99.8%].

Table 2 also shows that for GAM classification by mothers, prototype 3 demonstrated good sensitivity (90.7% [95% CI 87.4%; 93.7%]) and specificity (96.2% [95% CI 94.6%; 97.5%]). Younden’s *J* for GAM classification by mothers with prototype 3 was 86.8% [95% CI 83.2%; 90.1%] compared to the “gold standard”. However the sensitivity, specificity and agreement for GAM classification by mothers with the MUAC insertion tape was better: the sensitivity was 99.1% [95% CI 98.0%; 100.0%], the specificity was 96.5% [95% CI 95.0%; 97.7%] and Younden’s *J* was 95.6% [95% CI 93.7%; 97.2%]. The difference in Younden’s *J* for GAM classification by mothers using the prototype 3 compared with using the MUAC insertion tape is statistically significant: 86.8% [95% CI 83.2%; 90.1%] versus 95.6% [95% CI 93.7%; 97.2%] respectively.

The better performance of the MUAC insertion tape for GAM classification was also reflected in the results of the clinic staff. GAM classification by clinic staff using prototype 3 demonstrated good sensitivity (91.8% [95% CI 88.9%; 94.6%]) and specificity (97.2% [95% CI 95.8%; 98.3%]). Youden’s *J* for GAM classification by clinic staff with prototype 3 was 89.0% [95% CI 85.6%; 92.0%]. However both sensitivity and agreement for GAM classification by clinic staff with the MUAC insertion tape was significantly better: the sensitivity was 98.0% [95% CI 96.4%; 99.4%] and Younden’s *J* was 95.4% [95% CI 93.4%; 97.2%].

The study also sought to gather information on preference with regards to the devices used. Table 3 demonstrates that a higher proportion of mothers (33.3%) preferred prototype 3 to the other devices. The majority of clinic staff (70.7%) preferred the MUAC insertion tape.

**Table 3:** Device preferences for mothers and IMAM clinic staff

	Device			
	1	2	3	MUAC tape
Mothers	290 (27.9%)	156 (15.0%)	347 (33.3%)	247 (23.8%)
IMAM clinic staff	85 (07.9%)	58 (05.6%)	164 (15.8%)	735 (70.7%)

## Discussion

The results of this study indicate that, although the “Click-MUAC” devices performed well, the improved MUAC insertion tape performed best for mothers and caregivers classifying the nutritional status of their own children. The study team reported that mothers were concerned about pinching their child’s skin with devices 1 and 2 and this may have affected the ability of mothers to latch these devices properly. This may explain why the sensitivity is more reduced and to the same degree with these two devices (93.7% for device 1 and 93.8% for device 2). Sensitivity is less reduced with device 3. Given the nature of the design (similar to that of a tape) sensitivity in this case would have been affected by under-tensioning. This may have been due to the thickness of the plastic which may have compromised the device’s flexibility and hindered the mother’s ability to tension it properly. The tail of the tape in device 3 may also have been too short or too slippery for mothers to get a good grip.

The results for sensitivity of SAM classification by mothers with the MUAC tape are higher than those previously reported in the Blackwell et al. study [14] which demonstrated that mothers had a sensitivity for the classification of their child’s nutritional status of 73% and 90% respectively for SAM and GAM. It is possible that the high sensitivity reported in this study for both SAM and GAM classification by mothers with the MUAC insertion tape is due to the use of an improved MUAC tape design which has a number of modifications as compared to the conventional MUAC tape design, as illustrated in Fig. 2. It is also possible that the method of demonstration of the use of the tape to the mothers led to improved sensitivity. In the Blackwell et al. study [14] the use of the MUAC tape was demonstrated to the whole participating village. In this study however, mothers were provided with a one-on-one demonstration on the use of the MUAC insertion tape and the “Click-MUAC” devices by a member of the study team. There are some limitations to the study which could have biased the results presented in this paper. The study was only partially blinded and there may have been potential demonstration bias. It may also have been the case that mothers got better at fitting the devices as they worked through them, which could have influenced the sensitivity of the final device (the MUAC insertion tape). It is recommended that any similar study, conducted in the future, randomised the order of use of the prototypes to avoid this. The study did not collected further qualitative information regarding the preference of mothers for each of the devices. It is possible that the mothers preferred device 3 as it had the possibility to screen for both SAM and MAM but was sturdier and simpler to use than device 4 (uniMUAC tape). Clinical staff may have preferred device 4 (uniMUAC tape) because it enabled them to quantify the MUAC, rather than have a simple binary classification. Clinical staff may also have preferred device 4 (uniMUAC tape) because it more closely resembled the tape in current use (UNICEF MUAC tape).

## Conclusion

The results of this study demonstrate that although the “Click-MUAC” devices performed well, a well-designed MUAC insertion tape remains the best means to support mother-led MUAC screening. The MUAC insertion tape is also less costly to produce and may therefore be better suited to supporting larger-scale mother-led MUAC screening initiatives.

The results of this study provide strong evidence to support the ability of mothers to perform sensitive and specific measurements of their child’s MUAC. With an improved MUAC tape design and adequate minimal training, low MUAC children can be reliably identified by their mothers and caregivers. Given the potential for mother/caregiver MUAC screening to improve community case detection, early care-seeking behaviours and acute malnutrition treatment coverage, the approach should become central to efforts to scale-up acute malnutrition treatment globally.

## Abbreviations

AMREF: African Medical and Research Foundation
CHMT: County Health Management Team
CHV: Community Health Volunteer
CHW: Community Health Worker
CMAM: Community Management of Acute Malnutrition
ESRC: Ethics and Scientific Review Committee
GAM: Global Acute Malnutrition
IMAM: Integrated Management of Acute Malnutrition
MAM: Moderate Acute Malnutrition
MUAC: Mid-Upper Arm Circumference
OTP: Out-patient Therapeutic Programme
SAM: Severe Acute Malnutrition
uniMUAC: Universal Mid-Upper Arm Circumference
